# Assimilated Rank in the Navy

**Published:** 1850-06

**Authors:** W. Pierson

**Affiliations:** Rec. Sec. of Med. Soc. N. Jersey; New Brunswick, N. J.


### ASSIMILATED RANK IN THE NAVY.

At the annual meeting of the Medical Society of New Jersey, held at New Brunswick, May 14th, 1850, the following Preamble and Resolutions were adopted, viz:—

Whereas, it is a manifest duty, that organized medical bodies should exercise a proper influence for the protection of the rights of such regular members of the Profession as are necessarily detached from the great body of their brethren; and, whereas, many of the Medical Officers included in the military organizations of the country, are placed in this condition; and whereas, we have heard with regret, that there is a disposition on the part of a portion of the naval service, to deprive medical men connected with that Department, of the benefits arising from an assimilated rank, conferred by a general order of a late Secretary of the navy. Therefore be it

*Resolved,* That the "New Jersey State Medical Society" regards with much pleasure the successful efforts of the "Navy Boards," in raising the standard of Literary and Medical knowledge, for an admission to their ranks.

*Resolved,* That this Society is also much pleased to learn, that in their system of examinations *the Diplomas* of the schools (which are now but too easily obtained) are wholly disregarded; and that the moral character of the candidate, and his scientific and professional attainments, are his only passports to the medical corps of the navy.

*Resolved,* That this Society cannot look with indifference on any attempt to depress or degrade a whole class of public officers, belonging to a liberal profession, and so indispensable in the proper organization of the navy of their country.

*Resolved*, That as a well defined "Assimilated Rank," has been assigned to Medical officers of the Army, by an act of Congress, dated Feb. 11th, 1847, this Society cannot believe, that an invidious distinction will be made, between the *Medical Departments* of the Public service; but, that the National Legislature will protect the Surgeons and assistant Surgeons in their just claims to a *Nominal Rank*, or a *social* position, as respectable among the other grades of the Navy, as the *Medical Staff* of the Army now enjoy *by law*, in relation to their *brethren of the Line*, in that service.

*Resolved*, That a copy of these resolutions be forwarded to the Secretary of the Navy, through the Chief of the Medical Department; and also that a copy be forwarded to the chairman of the Naval Committee, in each house of Congress.

W. Pierson, M. D., Rec. Sec. of Med. Soc. N. Jersey.
*New Brunswick, N. J., May 14th*, 1850.

